# The effectiveness of workplace nutrition and physical activity interventions in improving productivity, work performance and workability: a systematic review

**DOI:** 10.1186/s12889-019-8033-1

**Published:** 2019-12-12

**Authors:** Aikaterini Grimani, Emmanuel Aboagye, Lydia Kwak

**Affiliations:** 1 Unit of Intervention and Implementation Research for Worker Health, Institute of Environmental Medicine, Karolinska Institutet, Nobels väg 13, Box 210, SE 171 77 Stockholm, Sweden; 2 Warwick Business School, University of Warwick, Coventry, UK

**Keywords:** Workplace health promotion interventions, Nutritional interventions, Fitness programs, Work-related outcomes, Absenteeism, Risk of bias, Randomized controlled trials, Non-randomized controlled study designs

## Abstract

**Background:**

Healthy lifestyles play an important role in the prevention of premature death, chronic diseases, productivity loss and other social and economic concerns. However, workplace interventions to address issues of fitness and nutrition which include work-related outcomes are complex and thus challenging to implement and appropriately measure the effectiveness of. This systematic review investigated the impact of workplace nutrition and physical activity interventions, which include components aimed at workplace’s physical environment and organizational structure, on employees’ productivity, work performance and workability.

**Methods:**

A systematic review that included randomized controlled trials and or non-randomized controlled studies was conducted. Medline, EMBASE.com, Cochrane Library and Scopus were searched until September 2016. Productivity, absenteeism, presenteeism, work performance and workability were the primary outcomes of our interest, while sedentary behavior and changes in other health-related behaviors were considered as secondary outcomes. Two reviewers independently screened abstracts and full-texts for study eligibility, extracted the data and performed a quality assessment using the Cochrane Collaboration Risk-of-Bias Tool for randomized trials and the Risk-of-Bias in non-randomized studies of interventions. Findings were narratively synthesized.

**Results:**

Thirty-nine randomized control trials and non-randomized controlled studies were included. Nearly 28% of the included studies were of high quality, while 56% were of medium quality. The studies covered a broad range of multi-level and environmental-level interventions. Fourteen workplace nutrition and physical activity intervention studies yielded statistically significant changes on absenteeism (*n* = 7), work performance (*n* = 2), workability (*n* = 3), productivity (*n* = 1) and on both workability and productivity (*n* = 1). Two studies showed effects on absenteeism only between subgroups.

**Conclusions:**

The scientific evidence shows that it is possible to influence work-related outcomes, especially absenteeism, positively through health promotion efforts that include components aimed at the workplace’s physical work environment and organizational structure. In order to draw further conclusions regarding work-related outcomes in controlled high-quality studies, long-term follow-up using objective outcomes and/or quality assured questionnaires are required.

**Trial registration:**

Registration number: PROSPERO CRD42017081837.

## Background

Physical inactivity and unhealthy eating behaviors are responsible for a substantial economic burden including lost productivity, which arises from two sources: absenteeism (time away from work due to illness or disability) and presenteeism (reduced productivity while at work) [1–4]. The workplace is an opportune setting for health promotion and for reaching a large part of the working population, including those individuals that are often difficult to reach, such as young men with lower social socioeconomic status. Promotion of physical activity and healthy nutritional behavior at the workplace could be an integrated initiative that improves worker health and enhances business performance [5]. According to the existing literature, workplace physical activity and nutrition interventions that involved counseling, education and on-site group activities have generally shown significant changes in employee sedentary and eating behavior, improving physical and mental health and a positive return on investment by reducing health care costs as well as overall absenteeism [6–8].

To date, several reviews have summarized the effectiveness of physical activity and nutritional workplace interventions [5, 7–9]. The majority of these reviews focus on health-related outcomes. Only few systematic reviews have focused on work-related outcomes, such as productivity and absenteeism [7, 9–11], and show that existing evidence is unclear and insufficient. Literature on the cost-effectiveness of workplace physical activity and nutrition interventions highlight the need for high-quality economic evidence, as the number of economic evaluation studies is limited and lacks methodological quality [6, 12, 13]. Knowledge on the economic and work-related outcomes of workplace health promotion interventions is of great importance for decision making that could further encourage investments in physical activity and nutritional strategies [14]. This is especially valuable due to limited resources forcing workplaces to choose between several intervention targets.

According to the Socio-ecological model, health behavior is a result of the interaction between the individual and the environment [15]. Promoting a change in physical activity and dietary behavior at the workplace should therefore include changes in workplace’s physical environment and organizational structure [16]. Exposure to changes in workplace’s physical environment (e.g. availability of healthy foods in vending-machines) and organizational structure (e.g. policies) can in addition facilitate behavior change in large segments of the working population [16, 17]. The evidence regarding the impact of those environmental and organizational changes on work-related outcomes is conflicting or insufficient, though [7]. Few reviews on the effectiveness of workplace physical activity and nutrition interventions have however reported on the effectiveness of intervention components aimed at changes in the workplace physical environment and/or organizational structure [7, 9, 10, 18].

Given the above, there is a growing literature on the evaluation of the effectiveness of workplace physical activity and nutrition interventions, however, there is still a gap regarding the work-related outcomes. In order to fill the gaps and shortcomings previously identified in the literature, the aim of the present systematic review was to investigate the impact of workplace physical activity and nutrition interventions, that include components aimed at the workplace’s physical environment and/or organizational structure, on employees productivity, work performance and workability.

## Methods

This systematic review was registered in the International Prospective Register of Systematic Reviews (with Registration number CRD42017081837) and adhered to the Preferred Reporting Items for Systematic Reviews and Meta-Analyses (PRISMA) statement [19, 20] and the Assessment of Multiple Systematic Reviews (AMSTAR) guidelines [21].

### Search strategy and inclusion criteria

A two-step search strategy has been followed. Firstly, a comprehensive literature search in Medline, Cochrane Library and PROPSERO was conducted, using Medical Subject Headings (MeSH) terms and relevant keywords, in order to identify previous systematic reviews with resembling objective as in this search regarding the impact of workplace nutrition and physical activity interventions on productivity, work performance and workability [7, 9, 18]. The MeSH is a controlled vocabulary for describing various biomedical topics which has been shown to greatly facilitate document retrieval [22]. Studies were identified from the reference lists of systematic reviews which met the inclusion criteria and were included in the review. In addition, Google Scholar search engine was searched.

Since the most recent systematic reviews with resembling objective had captured relevant studies until the year 2014 [7, 9, 18], an update search strategy following PICOS was also developed. The PICOS tool focuses on the Population, Intervention, Comparison, Outcome and Study design of an article. It is a framework designed to make the process of the literature searching more rapid and accurate, limiting the number of irrelevant articles [23]. Two experienced university librarians (CG, SG) developed the search strategy (Additional file 1) based on the provided PICOS. The search was conducted in four electronic databases: Medline, EMBASE.com, Cochrane Library and Scopus and covered year 2015 to September 2016.

The search strategies included studies conducted in High Income Countries (HICs), due to the different Occupational Health and Safety (OHS) context between the developed and the developing countries [14]. Searches were restricted to include studies with healthy adults over 18 years of age working in a full- or part-time capacity, and written in English language. Furthermore, studies which constitute “grey literature”, such as editorials, letters, working papers, reports and reviews were excluded.

Any workplace nutrition and physical activity intervention examining the organizational-, environmental- or multi-level effect on work-related outcomes such as productivity, absenteeism, presenteeism, work performance and workability was included. There was no restriction on the length of the intervention in order to study the short-term as well as the long-term effects of interventions. The research studies were either randomized controlled trials (RCTs) or non-randomized controlled study designs (NRSs) with a predefined control group or comparator group. Schelvis, Hengel [24] strongly suggest researchers who conduct systematic reviews to include studies applying alternative research designs such as NRSs. There were no restrictions on the type of comparator used in the study. The search process was done in accordance with the recommendations of the Cochrane Collaboration [25].

### Selection strategy

The screening process of abstracts and titles of systematic reviews was conducted by one reviewer. The systematic reviews were included if they met the inclusion criteria. Subsequently a reference list was conducted containing the studies of the selected systematic reviews.

Three reviewers (AG, EA, LK) independently selected studies by screening abstracts and titles of intervention studies, based on the inclusion criteria. Subsequently, three groups of two reviewers (group 1: AG, EA; group 2: AG, LK; group 3: EA, LK) independently determined the eligibility of studies on the basis of a review of the full texts, using a predesigned criteria form (Additional file 1). Any disagreement about selection in included studies were solved through discussion or by involving a third reviewer who did not participate to the group (AG or EA or LK).

### Quality assessment

In order to evaluate the methodological quality of included studies, the Cochrane Collaboration Risk of Bias Tool (CCRBT) for RCTs [25] and the Risk of Bias in NRSs of interventions tool (ROBINS-I) were used [26, 27]. ROBINS-I is mainly distinct from CCRBT because of the randomization. For the first three domains, randomization, if properly implemented, protects against biases that arise before the start of intervention but not after. Therefore, there is substantial overlap for the last four domains between the two assessment tools [25, 26]. The Cochrane Collaboration is strongly encouraging all reviewers to use these tools to establish consistency and avoid discrepancies in assessment of methodological quality among all review groups.

Methodological quality of each study was independently evaluated by two review authors (AG, EA), using the assessment tools. Disagreements were resolved by discussion until consensus was reached. A senior expert (GB) contributed to the assessment procedure whenever considered to be necessary.

### Data extraction and evidence synthesis

To identify the key elements from existing guidelines and texts, as well as from relevant systematic reviews, we followed the same data extraction procedure as in a previous systematic review [28]. A data extraction form was developed, reviewed and refined by the authors to better capture the key aspects that are essential for evaluation, synthesis and presentation, ensuring the adequacy of the tool. The data extraction form included information on publication (title, authors, year), location, occupation / industrial sector of target population, number of participants, company size and type, type of study design, measurement time period, type and description of the intervention, studies’ outcomes, our primary and secondary outcomes. Three reviewers extracted the data (AG, EA, LK) from equal number of studies. It was not possible to conduct a meta-analysis due to the heterogeneity of study designs, populations, interventions, and outcomes. We provided a narrative synthesis of the findings from the effective included studies, structured around the level of intervention (i.e. organizational-, environmental-, multi-level) and the type of the intervention (nutrition, physical activity, both).

## Results

### Literature searches

**Fig. 1:**
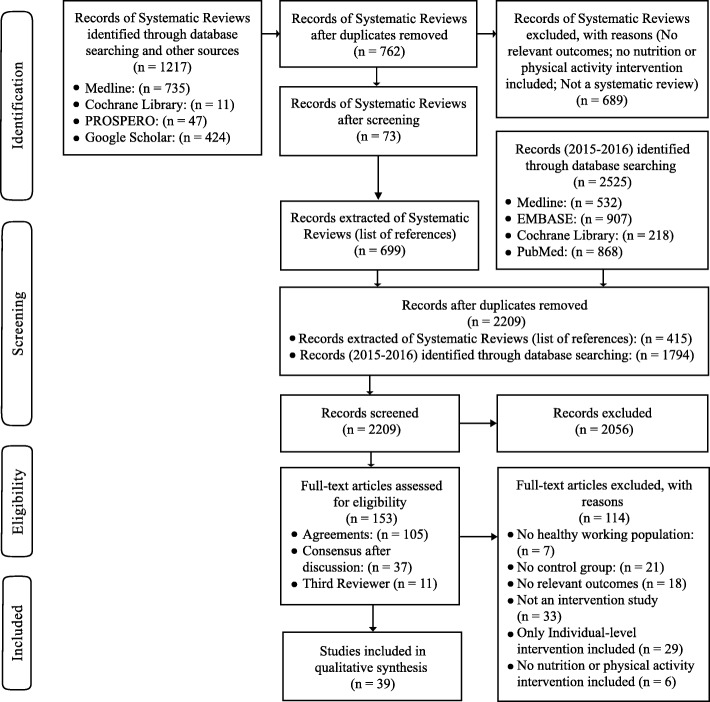
Flow Chart

Seventy three systematic reviews were assessed, resulting in a list of 699 RCTs and NRSs. After duplicates were removed (*n* = 284), a total of 415 citations remained for screening. After the update search, a total of 2209 citations were screened. One hundred and fifty three articles retrieved in full text, and 39 of them fulfilled the inclusion criteria (see Fig. 1).

### Description and characteristics of included studies

Nineteen included studies were RCT, while 20 included studies used NRS design, such as quasi-experimental controlled study design and pretest/ posttest controlled study design. Eighteen studies were carried out in the United States, seventeen studies in Europe and four studies in Australia. The studies included working populations from the following sectors: health care provider or insurance, services and administration, manufacturing, communication, education and multiple sectors. Sample sizes ranged from 25 to 155,543 employees (249,175 in total; mean sample size: 6557; median sample size: 407.5). Seven studies were conducted in the 1990s, nine studies were conducted during the following decade, while twenty three of the included studies were published after 2010. The follow-up period ranged from 3 months to 8 years, with upwards of 12 months as the most common duration for 22 studies. The studies reported on physical activity (*n* = 22), nutrition (*n* = 1) or both physical activity and nutrition (*n* = 16) interventions. The studies addressed either environmental-level (*n* = 4) or multi-level interventions (*n* = 35). The multi-level interventions were organizational- and individual-level interventions (*n* = 14), environmental- and individual-level interventions (*n* = 10) or environmental-, organizational- and individual-level interventions (*n* = 11). The included intervention studies targeted work-related outcomes such as absenteeism, presenteeism, work performance, workability and productivity and diverse types of health-related outcomes, such as sedentary behavior, physical activity, musculoskeletal comfort, weight loss and health risks. Two environmental-level [29, 30] and three multilevel intervention studies [31–33] aimed either at physical activity or nutrition measured work performance using subjective and objective measurements. An environmental-level [34] and two multilevel intervention studies [35, 36] aimed at physical activity measured workability using Work Ability Index (WAI). Six multilevel intervention studies [37–42] aimed either at physical activity or physical activity and nutrition measured productivity using objective and subjective measurements, such as HPQ (Health & Work Performance Questionnaire), WPAIQ (Work Productivity and Activity Impairment Questionnaire), Work Limitations Questionnaire (WLQ). Fourteen multilevel intervention studies aimed either at physical activity or physical activity and nutrition measured absenteeism. Nine of them used objective measurements (company records) [43–51], while five studies used subjective measurements [52–56]. An environmental-level [57] and ten multilevel intervention studies [58–67] aimed either at physical activity or physical activity and nutrition measured multiple work-related outcomes using objective and subjective measurements.

Table 1 presents the characteristics of the included studies (*N* = 39) and the interventions they studied, while an overall description of the interventions and details of the study designs are presented in Additional file 2. The studies were stratified according to the level of intervention and the type of the intervention. Eight categories were compiled: environmental-level interventions aimed at physical activity (*n* = 4); organizational- and individual-level interventions aimed at physical activity (*n* = 7), at nutrition (*n* = 1) and at both physical activity and nutrition (*n* = 6); environmental- and individual-level interventions aimed at physical activity (*n* = 7) and at both physical activity and nutrition (*n* = 3); environmental-, organizational- and individual-level interventions aimed at physical activity (*n* = 4) and at both physical activity and nutrition (*n* = 7).

**Table 1:** Characteristics of the included studies (*N* = 39) and the interventions they studied. An extended table (Additional file 2) is available in an online annex. [RCT = Randomized controlled trial; NRS=Non-randomized controlled study]

Categories	Variable	Number of studies
Level of intervention	Environmental	4 [29, 30, 34, 57]
	Multi-level: Organizational & Individual	14 [31, 33, 36, 40, 42, 43, 50, 51, 53–55, 60, 65, 66]
	Multi-level: Environmental & Individual	10 [32, 35, 37–39, 56, 58, 62–64]
	Multi-level: Environmental, Organizational & Individual	11 [41, 44–49, 52, 59, 61, 67]
Behavioral target of intervention	Physical Activity & Nutrition	16 [40, 42–50, 52, 55, 56, 60, 63, 64]
	Physical activity	22 [29–32, 34–39, 41, 51, 53, 54, 57–59, 61, 62, 65–67]
	Nutrition	1 [33]
Continent	Australia	4 [29, 38, 59, 61]
	USA	18 [30, 31, 33, 37, 39, 40, 42–44, 46–48, 50, 52, 55, 56, 60, 62]
	Europe	17 [32, 34–36, 41, 45, 49, 51, 53, 54, 57, 58, 63–67]
Sector/ industry	Health care provider or insurance	9 [33, 35, 36, 40, 46, 51, 65–67]
	Services & administration	10 [29–31, 37, 41, 53, 54, 56, 57, 61]
	Manufacturing	4 [44, 49, 50, 52]
	Communication	2 [38, 60]
	Education	1 [43]
	Multiple sectors	13 [32, 34, 39, 42, 45, 47, 48, 55, 58, 59, 62–64]
Type of study	RCT	19 [30–32, 35–37, 39, 41, 42, 51, 55–58, 61–64, 66]
	NRS	20 [29, 33, 34, 38, 40, 43–50, 52–54, 59, 60, 65, 67]
Intervention duration	> 12 weeks	4 [31, 35, 39, 59]
	> 12 months	10 [29, 32, 34, 37, 38, 40, 53, 54, 58, 61]
	≤ 12 months	22 [30, 36, 41–44, 46–52, 55, 56, 60, 62–67]
	Not stated	3 [33, 45, 57]
Work-related outcomes	Absenteeism	14 [43–56]
	Work performance	5 [29–33]
	Workability	3 [34–36]
	Productivity	6 [37–42]
	Multiple work-related outcomes	11 [57–67]
Measurement of work-related outcomes	Objective measurement	12 [30, 31, 33, 43–51]
	Subjective measurement	22 [29, 32, 34–37, 39–42, 52–56, 58–61, 64, 65, 67]
	Both objective & subjective measurement	5 [38, 57, 62, 63, 66]
Other outcomes	Health-related or other outcomes	25 [29, 30, 32, 34, 36–44, 49, 53, 54, 56, 58–61, 63–65, 67]
	Not stated	14 [31, 33, 35, 45–48, 50–52, 55, 57, 62, 66]

### Quality assessment

The overview of risk of bias assessment of the 19 RCTs is summarized in Additional file 3. The majority of the studies were assessed with a low or unclear risk of bias (see Cochrane Collaboration’s recommendations). Studies conducted before 2011 were assessed with an unclear risk of bias [31, 32, 36, 41, 55, 56], while more recent studies were assessed either with low [35, 37, 39, 57, 61, 63, 64] or unclear [30, 42, 58, 62] or high risk of bias [51, 66].

The overview of risk of bias assessment of the 20 NRSs is summarized in Additional file 3. None of the studies discussed deviations from intended interventions. According to Cochrane Collaboration’s recommendations, if there is a lack of information in one or more key domains of bias it is difficult to extract safe conclusion on which to base a judgment about risk of bias, as a result of which these studies are classified as no information. Thus, the studies were judged taking into consideration all the pre-intervention and at-intervention domains except bias due to deviations from intended interventions. The intervention studies were judged as having moderate risk of bias, as the majority of them provide sound evidence for a NRS but cannot be considered comparable to a well-performed randomized trial. Older studies (before 2011) were rated as having either moderate risk of bias [33, 43, 46, 47, 50, 52, 60] or no information [44, 48], while more recent studies were rated as having either low [29, 53, 54, 59] or moderate risk of bias [34, 38, 40, 45] or no information [65, 67].

### Efficacy of workplace nutrition and/or physical activity interventions for work-related outcomes

Fourteen studies (4 RCTs and 10 NRSs) were evaluated as being effective regarding the work-related outcomes, while two more NRSs showed effects only between subgroups. However, only two studies were rated as having low risk of bias [35, 57]. The effective studies were stratified according to the level of intervention and the type of the intervention. Three categories were compiled and presented below: (i) physical activity interventions aimed at changes in the workplace physical environment (*n* = 3); (ii) nutrition and physical activity interventions aimed at changes at multi-levels of the workplace (organizational- and individual-level: *n* = 4; environmental-, organizational- and individual-level: *n* = 6); (iii) physical activity interventions aimed at changes at multi-levels of the workplace (organizational- and individual-level: *n* = 2; environmental- and individual-level: *n* = 1). The overview of effective interventions is summarized in Table 2.

**Table 2:** Effective interventions on work-related outcomes

			Characteristics	Intervention	Primary outcomes	Secondary outcomes	Quality
Environmental	Physical Activity	Ben-Ner, et al. [30]^b^	USA; RCT; 12 months; *N* = 409	Treadmill desk	Work performance (objective & subjective measurement)	Physical activity (objective measurement)	Unclear Risk of bias
		Coffeng, et al. [57]^a^	Netherlands; RCT; *N* = 412	Social& physical environmental intervention	Work performance (subjective measurement)	N/A	Low Risk of bias
		Gao, et al. [34]^b^	Finland; Quasi-experimental controlled study; 6 months; *N* = 45	Sit–stand workstations	Workability (subjective measurement)	Occupational sedentary time &musculoskeletal comfort (subjective measurement)	Moderate Risk of bias
Multilevel Organizational, Individual	Physical Activity & Nutrition	Aldana, et al. [43]^a^	USA; Quasi-experimental controlled study; 24 months; *N* = 6246	WCSD Wellness Program	Absenteeism (objective measurement)	N/A	Moderate Risk of bias
		Lahiri and Faghri [40]^b^	USA; NRS; 16 weeks; *N* = 72	Incentivized Behavioral Weight Management Program	Productivity (subjective measurement)	Weight loss	Moderate Risk of bias
		Loeppke, et al. [60]^b^	USA; NRS; 36 months; *N* = 543	Health enhancement program	Absenteeism (subjective measurement) *Significant results only between subgroups	Health risks (subjective measurement)	Moderate Risk of bias
		Schultz, et al. [50]^a^	USA; NRS; 36 months; *N* = 4189	Workplace Health Promotion Program	Absenteeism (objective measurement)	N/A	Moderate Risk of bias
	Physical Activity	von Thiele Schwarz and Hasson [66]^a^	Sweden; RCT; 12 months; *N* = 177	Physical Exercise (PE)	Workability (subjective measurement)	N/A	High Risk of bias
		von Thiele Schwarz, et al. [65]^b^	Sweden; quasi-experimental controlled study; 24 months; *N* = 202	Integration program	Workability &Productivity (subjective measurements)	Health promotion, Integration, Kaizen, health (self-reported measurements)	No Information
Multilevel Environmental, Individual	Physical Activity	Jakobsen, et al. [35]^a^	Denmark; RCT; 10 weeks; *N* = 200	WORK (workplace physical exercise)	Workability (subjective measurement)	N/A	Low Risk of bias
Multilevel Environmental, Organizational, Individual	Physical Activity & Nutrition	Bertera [44]^a^	USA; pretest/ posttest controlled study; 20 months; *N* = 43,888	Multicomponent program (with 41 intervention sites)	Absenteeism (objective measurement)	N/A	Moderate Risk of bias
		Bertera [52]^b^	USA; pretest/ posttest controlled study; 24 months; *N* = 14,279	Multicomponent program	Absenteeism (subjective measurement) *Significant results only between subgroups	Behavioral risk factors (subjective measurement)	No Information
		Braun, et al. [45]^a^	UK; quasi-experimental controlled study; *N* = 155,543	Better Health atWork Award (Silver Award)	Absenteeism (objective measurement)	N/A	Moderate Risk of bias
		Conrad, et al. [46]^a^	USA (Michigan); quasi-experimental controlled study; 24 months; *N* = 1449	HRA, screening, counseling& Go to Health intervention program	Absenteeism (objective measurement)	N/A	Moderate Risk of bias
			USA (Indiana); quasi-experimental controlled study; 8 years; *N* = 746	“Alive and Well” program& health check	Absenteeism (objective measurement)	N/A	Moderate Risk of bias
		Jones, et al. [47]^a^	USA; NRS; 36 months; *N* = 1893	LIVE FOR LIFE program	Absenteeism (objective measurement)	N/A	Moderate Risk of bias
		Knight, et al. [48]^a^	USA; NRS; 36 months; *N* = 4972	LIVE FOR LIFE program	Absenteeism (objective measurement)	N/A	No Information

a: Effective on primary outcome(s)

b: Effective on primary and secondary outcomes

#### Physical activity interventions aimed at changes in the workplace physical environment

Three environmental-level intervention studies yielded statistically significant increases in workability and work performance. Ben-Ner, Hamann [30] RCT, which included 409 participants from a American financial service company, provided treadmills for 12 months and measured work performance using objective and subjective measurements. Physical activity was also measured yielding statistically significant effect. The study was judged as having unclear risk of bias. Coffeng, Hendriksen [57] RCT, which included 412 participants from a Dutch financial service company, carried out a social and physical environmental intervention and measured its effectiveness on work performance using subjective measurement (IWPQ). The study was judged as having low risk of bias. Moreover, Gao, Nevala [34] NRS, which included 45 participants from a Finish University, provided sit-stand workstations and measured workability using subjective measurement (WAI). Occupational sedentary time and musculoskeletal comfort were also measured, yielding statistically significant effects. The study was judged as having moderate risk of bias.

#### Nutrition and physical activity interventions aimed at changes at multi-levels of the workplace

Three organizational- and individual-level intervention studies, which were conducted in USA, yielded statistically significant effects on absenteeism and productivity. These studies were judged as having moderate risk of bias. Aldana, Greenlaw [43] NRS, which included 6246 participants from education sector, conducted a 24-month wellness program and measured absenteeism using objective measurement. Lahiri and Faghri [40] NRS, which included 72 participants from 4 nursing home facilities, carried out a 16-week incentivized Behavioral Weight Management program and measured productivity using subjective measurement (WLQ). Weight loss was also measured, resulting in statistically significant effects. Schultz, Lu [50] NRS, which included 4189 participants from a manufacturing company, conducted a 36-month workplace health promotion program and measured absenteeism using objective measurement. An additional NRS, which included 543 participants from communication sector, conducted a 24-month health enhancement program and measured self-reported absenteeism and health risks. The study, which was judged as having moderate risk of bias, yielded statistically significant effects, however only between subgroups [60].

Six environmental-, organizational- and individual-level intervention studies yielded statistically significant reductions in absenteeism using objective measurements. Bertera [44] NRS, which was classified as no information, included 43,888 participants from an American manufacturing company and conducted a 20-month multicomponent program. Braun, Bambra [45] NRS, which was judged as having moderate risk of bias, included 155 participants from British public and private sector and conducted a Better Health at Work Award program. Conrad, Riedel [46] included two NRSs which were conducted in USA at Blue Cross Blue Shield companies and were judged as having moderate risk of bias. The first studies included 1449 participants and received a 24-month “Go to health” program with screening and counseling, while the second study included 746 participants who received an 8-year “Alive and Well” program with health check. Jones, Bly [47] and Knight, Goetzel [48] NRSs conducted the same 36-month “Live for Life” program. The former included 1893 American workers from Johnson & Johnson Company and was judged as having moderate risk of bias while the latter included 4972 employees from Duke University and was classified as no information. An additional NRS, which included 14,279 participants from an American manufacturing company, conducted a 24-month multicomponent program to measure self-reported absenteeism and behavioral risk factors. The study, which was judged as having moderate risk of bias, yielded statistically significant effects only between subgroups [52].

#### Physical activity interventions aimed at changes at multi-levels of the workplace

Two organizational- and individual-level intervention studies, which were conducted in Sweden, yielded statistically significant increases in workability and productivity. Von Thiele Schwarz and Hasson [66] RCT, which included 177 participants from a large public dental health care organization, used a 12-month physical exercise intervention to measure workability using subjective measurement (WAI). The study was judged as having high risk of bias. Moreover, von Thiele Schwarz, Augustsson [65] NRS, which included 202 participants from 12 hospital units, used a 24-month integration program to measure workability and productivity using subjective measurement (WAI & HPQ, respectively). Health promotion, integration, kaizen and health were also measured, resulting in statistically significant effects. Due to insufficient data, the study was classified as no information. Furthermore, an environmental- and individual-level RCT, which included 200 female healthcare workers from 3 Danish hospitals, conducted 10-week workplace physical exercise with coaching sessions and ergonomic counseling to measure workability using subjective measurement (WAI). The study, which was judged as having low risk of bias, yielded statistically significant effects [35].

## Discussion

### Main findings

This systematic review sought to evaluate the effectiveness of workplace health promotion interventions, aimed at physical activity and/or nutrition, on productivity, absenteeism, work performance and workability. Only multi-level interventions, with components aimed at the workplace organization and/or physical work environment were included. The most frequently evaluated interventions were multi-level initiatives that included components focused on individuals, such as counseling, combined with components focusing on workplace environmental and/or organizational levels. A total of 39 studies, 19 RCTs and 20 NRSs, were included. The majority of studies were conducted in USA and Europe. There was a substantial increase in relevant published studies from 2010 onwards. Nearly 28% of the included studies were high quality, while 56% were of medium quality. The medium quality of the included studies was due to missing information considering the domains of bias.

Most of the studies measured absenteeism rather than productivity. This was perhaps to be expected, given that absenteeism data are easily and objectively assessed using workplace personnel records. Productivity on the other hand is arguably a more complex construct to measure [9]. Presenteeism, going to work while sick, is a significant problem, which accounts for a larger proportion of productivity losses compared to absenteeism and needs to be considered. However, the presenteeism literature is young and heterogeneous, thus, there was only limited evidence of a relationship between physical activity and presenteeism. Nevertheless, existing studies suggest that encouraging employees to be more physically active and reducing sitting can reduce presenteeism [10, 40].

Due to the lack of studies that have evaluated the impact of workplace interventions on other work-related outcomes, such as workability and work performance, knowledge appears to be limited. It is therefore difficult to draw general conclusions about the effects of interventions on a particular outcome as only a few studies have been conducted. In addition, there is an inherent problem in how the various work-related outcomes are measured, as there is no so-called gold standard for these types of outcomes. Few of the included studies have used the same questionnaires, and in some cases the same questionnaires are used to measure different outcomes. For instance, the World Health Organization Health and Work Performance Questionnaire (WHO-HPQ) was used to measure productivity, presenteeism and work performance. This makes it difficult to compare the results of the studies and to draw general conclusions about the impact of the initiatives.

Finally, the knowledge about the impact of the effects is limited as many of the included studies have had too short follow-up times, such as 4 weeks. Changes in work-related outcomes may have occurred after the final follow-up measurements have been completed. It takes time for a health promotion effect to lead to an improvement in physical activity and/or eating habits, which in turn is expected to lead to an improvement in work-related outcomes.

Overall, fourteen interventions were evaluated as being effective. Seven of these showed significant reductions in absenteeism (6 studies with moderate risk of bias and 1 study with no information), two studies showed effects on work performance (1 study with unclear risk of bias and 1 study with low risk of bias), three studies showed effects on workability (1 study with low risk of bias, 1 study with high risk of bias and 1 study with moderate risk of bias), one study showed effects on productivity (moderate risk of bias) and one study showed effects on both workability and productivity (no information). Two more studies showed effects on absenteeism only between subgroups (1 study with moderate risk of bias and 1 study with no information).

### Strengths and limitations

One of the strengths of this systematic review is the comprehensive search strategy used across major electronic databases, that facilitated a more evidence-based approach to literature searching. Moreover, inclusion of study designs other than RCTs is important in evaluating complex interventions, such as workplace health promotion interventions, as the implementation of an RCT may be difficult and/or ethically inappropriate in practice. Each included study in this review was comprehensively selected, assessed, data extracted and quality assessed by two review authors independently to minimize potential biases in the review process. Another important strength is the use of two methodological quality assessment tools, both recommended by Cochrane Collaboration, to assess the risk of bias of the included RCTs and NRSs. A limitation of this review is that studies in languages other than English, as well as unpublished studies (“grey” literature) were excluded. As a consequence, some useful and relevant studies might have been missed. In addition, all included studies were from high-income countries, restricting the generalizability of the results. Furthermore, the heterogeneous study components rendered a meta-analysis impossible.

## Conclusions and implications for future research

The scientific evidence of the present systematic review shows that it is possible to influence work-related outcomes, especially absenteeism, positively through health promotion efforts that include components aimed at the workplace’s physical work environment and organisational structure. Those studies showing reductions in absenteeism, evaluated long-term effects of nutritional behaviour and physical activity interventions in large populations, using objective measurements. The results of two high-quality RCTs and medium-quality studies indicate that efforts aimed at the workplace’s organizational structure and/or physical work environment can yield a positive impact on productivity, work performance and workability. However, there is still a lack of sufficient evidence regarding the impact of interventions on productivity, work performance and workability and thus more research is needed. In order to draw further conclusions regarding work-related outcomes in controlled high-quality studies, long-term follow-up using objective outcomes and/or quality assured questionnaires are required.

These results are likely to be meaningful for workers, employers and the policymakers who are involved in decision-making. Stakeholders can implement many health promotion programs and need to set priorities. The lack of information or the insufficient estimations regarding the work-related outcomes can lead to a sub-optimal allocation of available resources, and thus to forgone benefits from other and more advantageous health promotion programs. Furthermore, given that individuals spend the majority of their waking hours at the workplace, workplaces are ideal locations to implement effective health and wellness interventions that can reduce the burden associated with productivity loss. This review can serve as a guide for effective interventions targeting physical activity and/or nutrition and the positive changes of work-related outcomes, helping to set out policy priorities within Occupational Health and Safety.

## Supplementary information

**Additional file 1.** Search strategy and form of eligibility criteria.

**Additional file 2.** Characteristics and results of included studies.

**Additional file 3.** Risk of bias in included studies.

## Data Availability

This paper is a systematic review of previously published data. All data generated or analysed during this study are included in this published article (and its Additional files).

## Abbreviations

AMSTAR: Assessment of Multiple Systematic Reviews
CCRBT: Cochrane Collaboration Risk of Bias Tool
Embase: Excerpta Medica Database
HICs: High Income Countries
HPQ: Health & Work Performance Questionnaire
IWPQ: Individual Work Performance Questionnaire
MEDLINE: Medical Literature Analysis and Retrieval System Online
MeSH: Medical Subject Headings
NRS: Non-randomized controlled study design
OHS: Occupational Health and Safety
PICOS: Population, Intervention, Comparison, Outcome, Study design
PRISMA: Preferred Reporting Items for Systematic Reviews and Meta-Analyses
RCT: Randomized Control Trial
ROBINS-I: Risk of Bias in non-randomized studies of interventions
WAI: Work Ability Index
WHO-HPQ: World Health Organization Health and Work Performance Questionnaire
WLQ: Work Limitations Questionnaire
WPAIQ: Work Productivity and Activity Impairment Questionnaire
