# Supplementary material for: The effectiveness of workplace nutrition and physical activity interventions in improving productivity, work performance and workability: a systematic review
Source: BMC Public Health. 2019 Dec 12;19:1676. doi: 10.1186/s12889-019-8033-1 (PMC6909496; doi:10.1186/s12889-019-8033-1)
Supplement: Supplementary file 1 — Additional file 1. Search strategy and form of eligibility criteria. [file 12889_2019_8033_MOESM1_ESM.docx]

**Search strategies**

| **Databases** | **Search terms** |
| --- | --- |
|  |  |
| Medline (OVID) | 1. Workplace/  2. Work/  3. (employee* or employer* or employment).ti,ab,kf.  4. ((worker* or job* or personnel or staff or colleague* or occupation*) adj3 (workplace* or work place* or worksite* or work site* or work location* or work setting* or company or companies or busines* or factory or factories or office)).ti,ab,kf.  5. Occupational health services/  6. (occupational* or work*).ti.  7. or/1-6  8. Weight Loss/  9. (weight loss or weight gain or weight maintenance or weight reduction).ti,ab,kf.  10. exp Eating/  11. exp Food habits/  12. Food preferences/  13. dietary intake.ti,ab,kf.  14. ((calor* or fat* or salt* or fiber or fibre or sugar* or fruit* or vegetable* or carbohydrate*) adj3 (intake or intaking or consume or consumes or consumption or consumed or diet*)).ti,ab,kf.  15. ((nutritious or nutrition* or healthy or balanced or good) adj3 (menu or meal* or lunch* or dinner* or snack* or breakfast* or eating)).ti,ab,kf.  16. ((diet* or food* or dietary or eating or snack*) adj3 (behaviour* or behavior* or choice* or habit* or pattern* or preference* or nutritious or health* or intervention*)).ti,ab,kf.  17. vending machine*.ti,ab,kf.  18. exp Sports/  19. exp Exercise/  20. (exercise* or sport* or running or aerobic* or jogging or dance or dancing or badminton or tennis or swim* or squash or pilates or spinning class* or step* class* or yoga or gym* or football or rugby or netball or cricket or bowling or tai chi or weight lifting or lifting weight or weightlifting or hiit).ti,ab,kf.  21. (physical* adj3 (activit* or inactiv*)).ti,ab,kf.  22. Sedentary Lifestyle/  23. (sedentary or fitness).ti,ab,kf.  24. ((climb* or "use" or "using" or walk* or "take" or "taking") adj2 stair*).ti,ab,kf.  25. ("active at work" or "active travel").ti,ab,kf.  26. (shower* adj3 (provid* or access* or facilit* or availabl*)).ti,ab  27. (("use" or "using" or tak*) adj3 (public transport* or bus* or train* or tram* or subway*)).ti,ab,kf.  28. (walk* or bik* or cycling or bicycling or bicycle* or commut*).ti,ab,kf.  29. Health Promotion/  30. ((health or wellness) adj3 (promotion or program* or campaign*)).ti,ab,kf.  31. ((subsid* or voucher*) adj3 (gym* or sport* or leisur* or swim* or exercis* or public transport* or bus* or train* or tram* or subway*)).ti,ab,kf.  32. or/8-31  33. Absenteeism/  34. ((disabilit* or sickness or work* or time) adj3 (absence or absenteeism or loss*)).ti,ab,kf.  35. Presenteeism/  36. (presenteism or presenteeism or sickness presence*).ti,ab,kf.  37. Work Performance/  38. ((work* or job or vocational) adj3 (abilit* or capacit* or efficienc* or performance)).ti,ab,kf.  39. physical work* capacity*.ti,ab,kf.  40. exp "Costs and Cost Analysis"/  41. (employer health cost* or economic consequence* or capital expenditure* or cost*).ti,ab,kf.  42. ((health care or healthcare) adj3 (economic* or expenditure* or sector or cost*)).ti,ab,kf.  43. or/33-42  44. 7 and 32 and 43  45. limit 44 to yr="2015 -Current" |
| EMBASE.com | 1. 'workplace'/exp  2. 'work'/exp  3. (employee* or employer* or employment):ab,ti  4. ((worker* OR job* OR personnel OR staff OR colleague* OR occupation*) NEAR/3 (workplace* OR 'work place*' OR worksite* OR 'work site*' OR 'work location*' OR 'work setting*' OR company OR companies OR busines* OR factory OR factories OR office)):ab,ti  5. 'occupational health service'/exp  6. (occupational* or work*):ti  7. #1 OR #2 OR #3 OR #4 OR #5 OR #7 OR #8  8. 'weight reduction'/exp  9. ('weight loss' OR 'weight gain' OR 'weight maintenance' OR 'weight reduction'):ab,ti  10. 'eating'/exp  11. 'feeding behavior'/exp  12. 'dietary intake':ab,ti  13. ((calor* or fat* or salt* or fiber or fibre or sugar* or fruit* or vegetable* or carbohydrate*) NEAR/3 (intake or intaking or consume or consumes or consumption or consumed or diet*)):ab,ti  14. ((nutritious or nutrition* or healthy or balanced or good) NEAR/3 (menu or meal* or lunch* or dinner* or snack* or breakfast* or eating)):ab,ti  15. ((diet* or food* or dietary or eating or snack*) NEAR/3 (behaviour* or behavior* or choice* or habit* or pattern* or preference* or nutritious or health* or intervention*)):ab,ti  16. 'vending machine*':ab,ti  17. 'sport'/exp  18. 'exercise'/exp  19. (exercise* or sport* or running or aerobic* or jogging or dance or dancing or badminton or tennis or swim* or squash or pilates or spinning class* or step* class* or yoga or gym*or football or rugby or netball or cricket or bowling or tai chi or ‘weight lifting’ or ‘lifting weight’ or weightlifting or hiit):ab,ti  20. (physical* NEAR/3 (activit* or inactiv*)):ab,ti  21. 'sedentary lifestyle'/exp  22. (sedentary or fitness):ab,ti  23. ((climb* OR 'use' OR 'using' OR walk* OR 'take' OR 'taking') NEAR/2 stair*):ab,ti  24. ('active at work' OR 'active travel'):ab,ti  25. (shower* NEAR/3 (provid* or access* or facilit* or availabl*)):ab,ti  26. (('use' OR 'using' OR tak*) NEAR/3 ('public transport*' OR bus* OR train* OR tram* OR subway*)):ab,ti  27. (walk* or bik* or cycling or bicycling or bicycle* or commut*):ab,ti  28. 'health promotion'/exp  29. ((health OR wellness) NEAR/3 (promotion OR program* OR campaign*)):ab,ti  30. ((subsid* or voucher*) NEAR/3 (gym* or sport* or leisur* or swim* or exercis* or ‘public transport*’ or bus* or train* or tram* or subway*)):ab,ti  31. #11 OR #12 OR #13 OR #14 OR #15 OR #16 OR #17 OR #18 OR #19 OR #20 OR #21 OR #22 OR #23 OR #24 OR #25 OR #26 OR #27 OR #28 OR #29 OR #30 OR #31 OR #32 OR #33 OR #34  32. 'absenteeism'/exp  33. ((disabilit* or sickness or work* or time) NEAR/3 (absence or absenteeism or loss*)):ab  34. 'presenteeism'/exp  35. (presenteism or presenteeism or sickness presence*):ab,ti  36. 'job performance'/exp  37. ((work* or job or vocational) NEAR/3 (abilit* or capacit* or efficienc* or performance)):ab,ti  38. 'physical work* capacity*':ab,ti  39. 'cost'/exp  40. ('employer health cost*' OR 'economic consequence*' OR 'capital expenditure*' OR cost*):ab,ti  41. (('health care' or healthcare) NEAR/3 (economic* or expenditure* or sector or cost*)):ab,ti  42. #36 OR #37 OR #38 OR #39 OR #40 OR #41 OR #42 OR #43 OR #44 OR #45  43. 7 and 31 and 42  44. #48 AND (2015:py OR 2016:py OR 2017:py) |

| Scopus (Elsevier) | ( ( TITLE-ABS-KEY ( employee* OR employer* OR employment ) ) OR ( TITLE-ABS-KEY ( ( worker* OR job* OR personnel OR staff OR colleague* OR occupation* ) W/3 ( workplace* OR "work place*" OR worksite* OR "work site*" OR "work location*" OR "work setting*" OR company OR companies OR busines* OR factory OR factories OR office ) ) ) OR ( TITLE ( occupational* OR work* ) ) )  AND  ( ( TITLE-ABS-KEY ( ( subsid* OR voucher* ) W/3 ( gym* OR sport* OR leisur* OR swim* OR exercis* OR "public transport*" OR bus* OR train* OR tram* OR subway* ) ) ) OR ( ( TITLE-ABS-KEY ( "weight loss" OR "weight gain" OR "weight maintenance" OR "weight reduction" ) ) OR ( TITLE-ABS-KEY ( "dietary intake" ) ) OR ( TITLE-ABS-KEY ( ( calor* OR fat* OR salt* OR fiber OR fibre OR sugar* OR fruit* OR vegetable* OR carbohydrate* ) W/3 ( intake OR intaking OR consume OR consumes OR consumption OR consumed OR diet* ) ) ) OR ( TITLE-ABS-KEY ( ( nutritious OR nutrition* OR healthy OR balanced OR good ) W/3 ( menu OR meal* OR lunch* OR dinner* OR snack* OR breakfast* OR eating ) ) ) OR ( TITLE-ABS-KEY ( ( diet* OR food* OR dietary OR eating OR snack* ) W/3 ( behaviour* OR behavior* OR choice* OR habit* OR pattern* OR preference* OR nutritious OR health* OR intervention* ) ) ) OR ( TITLE-ABS-KEY ( "vending machine*" ) ) OR ( TITLE-ABS-KEY ( exercise* OR sport* OR running OR aerobic* OR jogging OR dance OR dancing OR badminton OR tennis OR swim* OR squash OR pilates OR "spinning class*" OR step* class* OR yoga OR gym* OR football OR rugby OR netball OR cricket OR bowling OR "tai chi" OR "weight lifting" OR "lifting weight" OR weightlifting OR hiit ) ) OR ( TITLE-ABS-KEY ( physical* W/3 ( activit* OR inactiv* ) ) ) ) OR ( ( TITLE-ABS-KEY ( sedentary OR fitness ) ) OR ( TITLE-ABS-KEY ( ( climb* OR "use" OR "using" OR walk* OR "take" OR "taking" ) W/2 stair* ) ) OR ( TITLE-ABS-KEY ( "active at work" OR "active travel" ) ) OR ( TITLE-ABS-KEY ( shower* W/3 ( provid* OR access* OR facilit* OR availabl* ) ) ) OR ( TITLE-ABS-KEY ( ( "use" OR "using" OR tak* ) W/3 ( "public transport*" OR bus* OR train* OR tram* OR subway* ) ) ) OR ( TITLE-ABS-KEY ( walk* OR bik* OR cycling OR bicycling OR bicycle* OR commut* ) ) OR ( TITLE-ABS-KEY ( ( health OR wellness ) W/3 ( promotion OR program* OR campaign* ) ) ) ) )  AND  ( ( TITLE-ABS-KEY ( ( disabilit* OR sickness OR work* OR time ) W/3 ( absence OR absenteeism OR loss* ) ) ) OR ( TITLE-ABS-KEY ( presenteism OR presenteeism OR "sickness presence*" ) ) OR ( TITLE-ABS-KEY ( ( work* OR job OR vocational ) W/3 ( abilit* OR capacit* OR efficienc* OR performance ) ) ) OR ( TITLE-ABS-KEY ( "physical work* capacity*" ) ) OR ( TITLE-ABS-KEY ( "employer health cost*" OR "economic consequence*" OR "capital expenditure*" OR cost* ) ) OR ( TITLE-ABS-KEY ( ( "health care" OR healthcare ) W/3 ( economic* OR expenditure* OR sector OR cost* ) ) ) )  AND  ( LIMIT-TO ( PUBYEAR , 2016 ) OR LIMIT-TO ( PUBYEAR , 2015 ) ) AND ( EXCLUDE ( DOCTYPE , "cp" ) OR EXCLUDE ( DOCTYPE , "cr" ) ) |
| --- | --- |

**Form of eligibility criteria**

| References |  | Does the paper include a worksite health promotion intervention? | Is the paper aimed at nutrition and/ or physical activity? (If yes report: N or PA or Both) | Does the paper include healthy working population? | Does the paper include an organizational and/ or environmental level intervention? (If yes report O or E or Both) | Does the paper include any primary outcomes? (If yes report P (or absenteeism or presenteeism) or WP or WA) | Does the paper include a control group? | **Results** | Study Design (observational, RCT, NRS, etc.) | Comments |
| --- | --- | --- | --- | --- | --- | --- | --- | --- | --- | --- |
|  | Reviewer 1 | Yes | Yes | Yes | Yes | Yes | Yes | **include** |  |  |
|  |  | No | No | No | No | No | No | **exclude** |  |  |
|  | Reviewer 2 | Yes | Yes | Yes | Yes | Yes | Yes | **include** |  |  |
|  |  | No | No | No | No | No | No | **exclude** |  |  |
|  | Reviewer 3 (if required) | Yes | Yes | Yes | Yes | Yes | Yes | **include** |  |  |
|  |  | No | No | No | No | No | No | **exclude** |  |  |
