# Supplementary material for: The effectiveness of workplace nutrition and physical activity interventions in improving productivity, work performance and workability: a systematic review
Source: BMC Public Health. 2019 Dec 12;19:1676. doi: 10.1186/s12889-019-8033-1 (PMC6909496; doi:10.1186/s12889-019-8033-1)
Supplement: Supplementary file 2 — Additional file 2. Characteristics and results of included studies. [file 12889_2019_8033_MOESM2_ESM.docx]

**The impact of worksite nutrition and physical activity interventions on productivity, work performance and work ability: A Systematic Review**

| Characteristics and results of included studies. [RCT=Randomized controlled trial; NRS=Non-randomized controlled study] | | | | | | | | |
| --- | --- | --- | --- | --- | --- | --- | --- | --- |
|  | | | Country; Industrial sector; Company size | Number of participants; Type of study design; Measurement time period | Description of the intervention and control condition | Primary outcomes | Secondary outcomes | Results |
| Environmental | Physical Activity | Alkhajah, et al. (29)^b^ | Australia; Academic office workers | N=32 (I=18, C=14); Quasi-experimental controlled study; 3 months (baseline: 1-week; follow-up: 3-month) | I: Ergotron WorkFit-S, Single LD Sit-Stand Workstation with brief verbal (2 min) and written instructions  C: control group (usual daily activities) | Work performance (self-reported using nine-item work performance scale) | Sedentary behavior (Objective measurement 24/7 using activPAL3 monitors) | No significant differences between the groups regarding work performance.  Significant decrease in sedentary behavior with participation in the intervention (I: 143 min / day on working hours (95% CI = -184, -102) & 97 min / day throughout the day (95% CI = -50) at 1 week follow-up; at 3-month follow-up (-137 min / day and -78 min / day, respectively). |
|  |  | Ben-Ner, et al. (30)^c^ | USA; financial service company | N= 409 (I1=17, I2=23, C=369); RCT; 12 months (baseline, weekly follow up) | I1: Walker 1 (received treadmills in June 2008)  I2: Walker 2 (received treadmills in late December 2008)  C: control group (did not receive treadmills) | Work performance* (objective measurement using company administrative records & subjective measurement using survey) | Physical activity (objective measurement using accelerometer) | Access to treadmill workstations resulted in improvement in overall work performance, and quality and quantity of work performance and in an increase in physical activity (p <0.05). |
|  |  | Coffeng, et al. (57)^a^ | Netherlands; financial service | N= 412 (I1=92, I2=118, I3=96, C=106); RCT; (baseline, 6-month and 12-month follow-up) | I1: social environmental intervention (group motivational interviewing (GMI)conducted by team leaders (3 times 90 minutes over 6 weeks & a booster session after 2 months))& physical environmental intervention (table tennis, sitting balls, lounge chairs, posters)  I2: social environmental intervention only  I3: physical environmental intervention only  C: control group (no intervention) | Absenteeism* (objective measurement using company records), Presenteeism* (self-report with the World Health Organization Health and Work Performance Questionnaire (WHO-HPQ)), Work performance* (self-report with the Individual Work Performance  Questionnaire (IWPQ)), Work engagement* (the Utrecht Work Engagement  Scale) | None reported | Significant differences between I1 & C and I2 & C regarding work performance at 12 months follow-up. (I1: Context Performance = 3.2 (95% CI -0.4, -0.1; P = 0.01); C: Context Performance=3.6).  (I2: task performance = 3.7 (95% CI 0.0, 0.4; p = 0.04); C: task performance = 3.6).  No significant results regarding absenteeism and presenteeism.  The interventions in the current form are not recommended  to be implemented to target the  work-related outcomes. |
|  |  | Gao, et al. (34)^c^ | Finland; University staff (researchers, teachers,  administrative workers, assistants, professors and  technical workers) | N=45 (I=24, C=21); Quasi-experimental controlled study; 6 months (baseline, 6-month follow-up) | I: sit–stand workstations  C: control group, traditional sitting workstations | Workability* (self-reported measurement using work ability index (WAI)) | Occupational sedentary time  Musculoskeletal comfort  (self-reported measurement using questionnaires) | Significant improvement of workability (p = .022).  Significant reduction in sitting time at sit–stand workstations compared to control workstations (−6.7% vs. 5.0%, p = .019).  Significant improvement of musculoskeletal comfort in the neck and shoulders  (p = .028). |
| Multilevel  Organizational, Individual | Physical Activity & Nutrition | Aldana, et al. (43)^a^ | USA; Education (90 schools) | N=6246 (I1=1264; I2=1407; C=3575); Quasi-experimental controlled study; 24 months (baseline:1997-2000; follow-up: 2001-2002) | Project: WCSD Wellness Program (11 different wellness programs: brighten your smile, holiday weight challenge, H_2_0 challenge, Tame the TV, March nutrition mystery, Mount Everest fitness challenge, test your rest, Ironman triathlon fitness challenge, Train your brain, Exercise for life, Buckle up America)  I1: 2-year participation in the intervention  I2: 1 year participation in the intervention  C: control group (no participation in the intervention) | Absenteeism* (objective measurement; was reported in hours by HR) | Health care costs (objective measurement; employee claims data) | Significant decrease in absenteeism with participation in the program (I1 = 14.3 days / year (95% CI = 0.70, 0.92; p = 0.000) and I2 = 15.1 days / year (95% CI=0.80, 1.05; p=0.019) compared to C (15.4 days / year); a cost savings of US$15.60 for every dollar spent on the program.  No significant differences in health care costs between the interventions and control group |
|  |  | Jeffery, et al. (55) | USA; 32 businesses | N= 1242 (I=597, C=645);RCT; 24 months (baseline, 2-year follow-up) | Healthy Worker Project (HWP)  I: 11 courses focused on smoking cessation & obesity  C: control group, no intervention | Absenteeism* (self-reported measurements) | None reported | A net reduction in absenteeism was associated with smoking programs but not with weight programs. |
|  |  | Lahiri and Faghri (40)^c^ | USA; 4 Nursing home facilities | N= 72 (I=35, C=37); NRS; 16 weeks (baseline, 28-week follow-up) | I: Behavioral Weight Management Program with incentives  C: Behavioral Weight Management Program without incentives | Productivity (self-reported measurement using Work Limitations Questionnaire (WLQ)) | Weight loss | Significantly higher net savings based on absenteeism in the I group compared to the C group.  Significant differences between the body weight reduction groups at 28-week follow-up (P <0.05). |
|  |  | Loeppke, et al. (60)^c^ | USA; Communication DIRECTV | N=543; NRS; 36 months (baseline, 24-month follow-up, 36-month follow-up) | I:health enhancement program  C: 2 control groups without health enhancement program. | Absenteeism  Productivity  Work performance (self-reported measurements using Health and Work Performance Questionnaire (HPQ) | Health risks(self-reported measurements using HRA) | No statistically significant changes between groups regarding work-related outcomes.  Significant decrease in absenteeism among I group.  Significant reduction in health risks at 24 months. |
|  |  | Schultz, et al. (50)^a^ | USA; manufacturing company | N= 4189 (I=2596, C=1593); NRS; 36 months (baseline; 12-month follow-up, 24-month follow-up, 36-month follow-up, 48-month follow-up, 60-month follow-up) | I: participation in at least one of the programs: HRA, on-site health screening, on-site & telephonic wellness programs, medical vouchers, nurse telephone counseling  C: control group, no intervention | Absenteeism* (objective measurements using workers' compensation absences& administrative records) | None reported | Significant differences between the groups regarding annual increases in absent days in follow-up (p <0.01).The average annual increase in absent days in I was 2.4 days / year compared to 3.6 days / year in C between 1995-2000. Participants in C increased their absent days by 1.2 days per year more than participants in I. |
|  |  | Terry, et al. (42) | USA; Large health care company and airline company | N= 631 (I1=259, I2=202, C=170); RCT; 24 months (baseline, 24-month follow-up) | I1:Traditional Health Improvement Program: seminars & interactive educational campaigns, health coaching for high risk workers (2 face to face & 11 telephone visits)  I2: Activated Consumer Program: health consumerism communications materials, group programs along with nurse-navigator coaching for high risk workers  C: control group, information on personal development topics | Productivity* (self-reported measurement using 10-scale item from Health & Work Performance Questionnaire (HPQ)) | Health risk status(self-reported using Personal Wellness Profile and clinical tests)  General health status (self-reported using a 5-scale question) | No significant differences between the groups regarding productivity in the follow-up. |
|  | Physical Activity | Dallat, et al. (53) | Northern Ireland; government officers | N= 406 (I=199, C=207);quasi-experimental controlled study; 12 weeks (baseline, 6-month follow up) | Physical Activity Loyalty (PAL) card scheme  I: physical activity with incentives (rewards for recorded minutes of physical activity using the PAL card)  C: control group, physical activity without incentives | Absenteeism (self-reported measurement using QOL &EQ-5D) | Physical activity (objective measurement using PAL card records & self-reported measurement using the Global Physical Activity  Questionnaire (GPAQ)) | No significant differences between the groups regarding physical activity, and absenteeism in follow-up. |
|  |  | Hunter, et al. (54) |  |  |  |  |  |  |
|  |  | Galinsky, et al. (31) | USA; service business | N = 51 (I = 21, C = 30); RCT (cross-over); 8 weeks | I: stretching exercises during conventional breaks (4 weeks) & supplementary breaks (4 weeks)  C: control group, no stretching exercises | Work performance* (objective measurement using electronic records) | Non reported | No significant results regarding work performance |
|  |  | von Thiele Schwarz, et al. (36)^b^ | Sweden; Large public dental health care organization | N= 177 (I1=62, I2=50, C=65); RCT; 12 months (baseline, 6-month follow-up, 12-month follow-up) | I1:physical exercise (two mandatory equal PE periods for a total 2.5 h/week)  I2: reduced work hours (reduction from 40 hours/week to 37.5 hours/week for full-time workers & proportional curtailment for part-time workers)  C: control group, no intervention | Workability* (self-reported measurement using Work Ability Index (WAI)) | Physical activity (self-reported measurement using two questions) | No significant differences between groups regarding workability.  Significant increase regarding physical activity in I1 compared with I2 (p = 0.024) and with K (p <0.001) in the follow-up. |
|  |  | von Thiele Schwarz and Hasson (66)^a^ |  |  |  | Workability*(self-reported measurement using Work Ability Index (WAI))  Absenteeism* (self-reported measurement using STEM Questionnaire)  Productivity* (objective measurements using administrative records (mean number of treated patients per therapist) | None reported | Significant differences between groups regarding workability (P <0.001). I1 group increased workability compared with the C group.  No significant differences between groups regarding absenteeism and productivity. |
|  |  | von Thiele Schwarz and Hasson (51) |  |  |  | Absenteeism*  (objective measurement using administrative records) | None reported | Absenteeism decreased in I1 & I2 (% change I1: -41.2; I2: -9.5) but increased among C (% change 52.9).  However no statistical tests have been performed. |
|  |  | von Thiele Schwarz, et al. (65)^c^ | Sweden; 12 hospital units | N= 202 (I=111, C=91); quasi-experimental controlled study; 24 months (baseline, 12-month follow-up, 24-month follow-up) | I: integration program (workshops and coaching)  C: control group, no intervention program | Workability* (self-reported using Work ability Index (WAI))  Productivity* (self-reported using Health and Work Questionnaire (HPQ))  Absenteeism* (self-reported) | Health promotion  Integration  Kaizen  Health  (self-reported measurements using questionnaires) | Significant improvement in workability and productivity.  No significant differences in absenteeism.  Significant increase in the health promotion, integration, Kaizen & health in the I compared with the C. |
|  | Nutrition | Trudeau, et al. (33) | USA; Insurance company | N= 618 (I=309,C=309); pretest/ posttest controlled study; several weeks (baseline, 15-month follow-up) | Connections program  I: health promotion & substance abuse prevention.  C: control group, no intervention. | Work performance* (objective measurement using company records) | None reported | No significant differences between groups regarding work performance in follow-up. |
| Multilevel  Environmental, Individual | Physical Activity & Nutrition | Meenan, et al. (56)^b^ | Hawaii; 31 Oahu hotels | N= 6958 (I1=3612, I2=3346); RCT; 24 months (baseline, 24-month follow-up) | Work, Weight and Wellness (3W)  I1:weight loss & obesity prevention program  I2:brief feedback on weight & lifestyle choices | Absenteeism (self-reported measurement) | BMI | No statistical results are described regarding changes in absenteeism.  Significant changes in BMI at 24 months follow-up p<0.05). |
|  |  | Van Dongen, et al. (63) | Netherlands; governmental  research institute employees | N= 257 (I1=3612, I2=3346); RCT; 12 months (baseline, 6-month follow-up, 12-month follow-up) | I: Mindful VIP intervention (mindfulness  training, e-coaching, and supporting elements (eg. fruit and vegetables,  lunch walking routes, a buddy system))  C: control group, no intervention | Workability* (self-reported using Work Ability Index (WAI))  Absenteeism (objective measurement using company records)  Presentreeism (self-reported using World  Health Organization Health and Work Performance Questionnaire (WHO-HPQ)) | Work engagement (self-reported using Utrecht Work  Engagement Scale)  General vitality (self-reported using RAND-36 Vitality Scale)  Job satisfaction (self-reported) | No significant differences in workability.  Significant adverse effect on work engagement was found (-0.19; 95%  CI: -0.38 to -0.01).  No significant differences in job satisfaction, general vitality. |
|  |  | van Wier, et al. (64) | Netherlands; 1 or more companies  with more than 100 workers | N= 523 (I=274; C=249); RCT; 18 months (waist & body: baseline, 18-month follow-up; presenteeism & absenteeism: every 3 months retrospectively (6 times over 18 months)) | I: environmental scan, assessing the workplace facilities to promote prevention of weight gain among employees (5 individual counselling sessions with Occupational Physicians (OP) with 20-30min duration & informational tools)  C: control group, usual care & health care advise by OP | Absenteeism(self-reported measurement using Productivity & Disease Questionnaire)  Presenteeism (self-reported measurement using World Health Organization Health & Work Performance Questionnaire (WHO-HPQ)) | waist circumference  body weight (objective measurement using tape and pedometer) | No significant differences between the groups regarding absenteeism at 18 months follow-up.  Significant differences between the groups regarding the presenteeism in 18-months follow-up. However no financial return was shown.  Unfavourable differences on waist circumference (+1.6 cm; 95% confidence interval CI: 0.27 to 2.90) and weight (+1.1 kg; 95% CI, 0.01 to 2.15). |
|  | Physical Activity | Carr, et al. (37)^b^ | USA; Office employees of alarge private company | N= 54 (I=27; C=27); RCT; 8 months (baseline, 16-week follow-up) | I: HP/HP group (ergonomic workstation optimization intervention;  three activity-promoting e-mails/ week and access to a seated active workstation)  C: comparison group, HPO group (ergonomic intervention and e-mails only). | Productivity (self-reported using World Health Organization Health & Work Performance Questionnaire (WHO-HPQ)) | Occupational sedentary and physical activity behaviors (objective measurements using ankle-worn accelerometer  (GENEActiv)) | No intervention effects for  Productivity.  The integrated  HP/HP intervention significantly increased occupational  physical activity when compared  with the non-integrated HPO group. |
|  |  | Chau, et al. (38)^b^ | Australia; Large telecommunications company | N= 31 (I=16; C=15); quasi-experimental controlled study; 19 weeks (baseline, 1-week follow-up, 4-week follow-up, 19-week follow-up) | I:sit-stand desk, brief training, and daily e-mail reminders to stand up more frequently  C: control group, no intervention | Productivity (objective measurement using company-specific objective metrics (e.g., hold time, talking time, absenteeism) and self-reported measurement) | Sitting &Physical activity (self-reported using ActivPAL or ActiGraph devices and self-report questionnaires) | No significant differences regarding productivity.  Significant reduction in sitting time (−100min per workday at 19-week follow-up), compared with the control group |
|  |  | Dalager, et al. (58)^b^ | Denmark; Office workers from 12 geographically different units | N= 356 (I1=72; I2=81; I3=55; I4=65; C=83); RCT; 20 weeks (baseline, 20-week follow-up) | I1: 1WS (one 60-min supervised session/week)  I2: 3WS (three 20-min supervised sessions/week)  I3: 9WS (nine 7-min supervised sessions/week)  I4: 3MS (three  20-min sessions/week with minimal supervision)  C: control group, no intervention | Workability*  Productivity*  (self-reported using questionnaires) | Self-rated health Musculoskeletal pain symptoms  (self-reported using questionnaires) | No significant differences regarding workability and productivity.  No change from baseline to  follow-up for self-rated health.  Significant decrease regarding musculoskeletal pain symptoms in all I groups compared with C. |
|  |  | Dutta, et al. (39)^b^ | USA; sedentary office workers (Caldrea Inc.) | N = 28; RCT; 4 weeks | I: 3 different models of desks (Workfit-S, Workfit-A, Workfit-D), ergonomic evaluation, weekly email reminder (replacing 50% of sitting with standing)  C: control group, performing usual work habits.  2-week "washout" period between intervention & control period without ergonomic desks & measurements. | Productivity (self-reported measurement using Work Productivity and Activity Impairment Questionnaire (WPAIQ) | Sedentary behavior (objective measurement using accelerometer & self-reported measurement using OSPAQ)  physical activity (objective measurement using accelerometer)  eating habits (self-reported) | No significant differences regarding productivity between intervention and control periods.  Sitting time significantly decreased by 21% (I: 95% CI 18% - 25%) &sedentary time by 4.8 min / working hours (I: 95% K.I 4.1-5.4 min / working hours) compared to C.  Calorie intake significantly decreased during the intervention period by 212 kcal / day (95% CI 45 to 379 kcal; p = 0.01). |
|  |  | Jakobsen, et al. (35)^a^ | Denmark; female healthcare workers from 18 departments at three hospitals | N = 200 (I=111; C=89); RCT; 10 weeks (baseline, 10-week follow-up) | I: WORK: workplace physical exercise  for 5x10 min/ week, up to 5 group-based coaching sessions &ergonomic counseling  C: HOME: home-based physical exercise during leisure time for 5x10 min/ week& ergonomic counseling. | Workability* (self-reported using Work Ability Index (WAI)) | None reported | Significant differences between groups regarding workability, which at follow-up was 1.1 (0.3 to 1.8)  higher in I compared with C. |
|  |  | Puig-Ribera, et al. (32)^b^ | Spain; Catalan University | N= 70 (I1=19, I2=25, C=26); RCT; 3 months (work performance: baseline, 1-week follow-up, 9-week follow-up; steps: baseline, 1-weel follow-up, 5-week follow-up, 9-week follow-up) | I1:Map with walking tips  within the area, emails with advice on  physical activity(15minutes/ working day)  I2:Advice on physical activity  (eg. lectures,  seminar, meeting) and e-mails.  C: control group, usual walking behavior | Work performance* (self-reported measurement using Work limitations Questionnaire (WLQ)) | Steps (Objective measurement using Yamax SW 200 pedometer) | No significant differences between the groups regarding work performance.  No significant results regarding the number of steps (only between subgroups: a significant increase in step counts (+659 steps/day; p<0.01) among participants classified as “Sedentary–Low active” at baseline). |
|  |  | Snetselaar, et al. (62) | Iowa; University of Iowa, 5 local businesses, and 1 regional business | N= 280 (I=138, C=142); RCT; 3 years (baseline, 1-year, 2-year follow-up) | Be Hipp program  I:interactive  participatory monthly group lunch sessions discussing about nutrition, physical activity, stress management, and ergonomics  C: control group, quarterly newsletters only | Absenteeism*(objective measurement using company records)  Work performance (self-reported using Work Limitations Questionnaire (WLQ)) | None reported | No statistical difference in absenteeism hours  between the I and C. |
| Multilevel  Environmental, Organizational, Individual | Physical Activity & Nutrition | Bertera (44)^a^ | USA; Large manufacturing company (60 departments) | N = 43888 (I = 29315, C = 14573); pretest/ posttest controlled study; 20 months (baseline, 2-year follow up) | I: (41 intervention sites) multicomponent program: training for site coordinators, a Health Promotion Activity Committee, Orientation and Publicity, Health Risk Appraisal, self-directed and group health education opportunities.  C: control group (19 control sites) no intervention | Absenteeism* (objective measurement using time  cards signed by supervisors) | None reported | Significant decrease in absenteeism/ employee  I = 0.7 days / year (95% CI = 0.3, 0.5) compared to C (0.3 days / year) in follow-up in 1986. Over two years 11726 fewer absent days among intervention departments versus control units. |
|  |  | Bertera (52)^c^ | USA; Large manufacturing company | N= 14279 (I=7178, C=7101);pretest/ posttest controlled study; 24 months (I: baseline 1984-1985; 2-year follow-up:1986-1988& C: baseline 1987 -1988) | I: multicomponent program: training of coordinators, health risk appraisals, on-site classes, safety meeting & self-help options, environmental changes, recognition.  C: control group, no intervention | Absenteeism* (self-reported measurement) | Behavioral risk factors (self-reported using 7-scale questionnaire) | Significant decrease (12%) in absenteeism at 24 months follow-up among high risk employees (4.1 days at baseline versus 3.6 days at follow-up; p <0.001).  Number and level of behavioral risk factors were improved. |
|  |  | Braun, et al. (45)^a^ | UK; 232 public & private organizations | N=155 543; quasi-experimental controlled study | Better Health atWork Award  I1: Bronze Award  I2: Silver Award  I3: Gold Award | Absenteeism* (objective measurement using company records) | None reported | A mean reduction in absenteeism of 0.26–1.6 days per employee per year.  Only the Silver Award scheme led to a significant decrease in absenteeism.  Estimated cost for the programme: £3 per absent day saved. |
|  |  | Conrad, et al. (46)^a^ | USA (Michigan);Blue Cross Blue Shield companies | N= 1449; quasi-experimental controlled study; 24 months (baseline, 2-year follow up) | I1:HRA, screening, counseling, Go to Health intervention program  I2:HRA, screening, counseling  I3:HRA  C: control group, no intervention | Absenteeism* (objective measurement using company records) | None reported | I1 participants significantly reduced their absent hours in 24 months follow-up compared to I2, I3 and C participants (p <0.05). |
|  |  |  | USA (Ohio); Blue Cross Blue Shield companies | N= 1448; quasi-experimental controlled study; 24 months (baseline, 1-year follow up) | I: 3 intervention programs “Health Builder” (health survey, counseling, physical activity, weight loss, stress, smoking)  C: control group  (each different program compared with a control group) | Absenteeism* (objective measurement using company records) | None reported | No significant differences between the groups regarding absenteeism in follow-up. |
|  |  |  | USA (Indiana); Blue Cross Blue Shield companies | N= 746(I1=181; I2=388; C=177); quasi-experimental controlled study; 8 years (baseline, 8-year follow up) | I1: health check & “Alive and Well” program  I2: health check only  C: control group no intervention | Absenteeism* (objective measurement using company records) | None reported | I1& I2 participants significantly reduced their absenteeism in follow-up compared to C participants. |
|  |  | Jones, et al. (47)^a^ | USA; Johnson & Johnson company | N= 1893 (I=1406, C=487); NRS; 36 months (baseline, 12-month follow-up, 24-month follow-up) | LIVE FOR LIFE program  I: smoking cessation, weight control, stress management, nutrition, education, fitness, blood pressure  C: control group, no intervention | Absenteeism* (objective measurement using company records) | None reported | Significant differences between the groups regarding the number of absent hours during 24 months follow-up. |
|  |  | Knight, et al. (48)^a^ | USA; Duke University | N= 4972 (I=3122, C=1850); NRS; 36 months (baseline, 12-month follow-up, 24-month follow-up, 36-month follow-up) | LIVE FOR LIFE program  I: smoking cessation, weight control, stress management, nutrition, education, fitness, blood pressure  C: control group, no intervention | Absenteeism* (objective measurement using university records) | None reported | Significant differences between the absent hours in 36-monthfollow-up (4.6 less absenteeism in I compared to C (95% CI: 0.2, 8.6; p <0.05). |
|  |  | Maes, et al. (49) | Netherlands; 3 sites of manufacturing company | N= 264 (I=134, C=130); Quasi-experimental pretest / post-test controlled study; 36 months (baseline, 12-month follow-up, 24-month follow-up, 36-month follow-up) | Healthier Work at Brabantia  I: physical activity & health education, nutrition, drug consumption, stress, smoking behavior, back pain, headaches; training in social skills & leadership, smoking policy in cafeteria, posters, videos, internal radio messages, newsletter articles, healthy food, incentives to promote participation in the program  C: control group, no intervention | Absenteeism* (objective measurement using administrative records) | Lifestyles, health risks, general stress reactions, working conditions (self-reported measurements) | No statistically significant absenteeism changes between groups (there were decreases in absenteeism 8.1% in the I group & 4.8% in the C group but no statistical analysis was reported).  No statistically significant effects on secondary outcomes. |
|  | Physical Activity | Healy, et al. (59)^b^ | Australia; Government agency for work & health | N= 43 (I=22, C=21); NRS; 4weeks (baseline, 4-week follow up) | I: (Stand up, Sit Less, Move More) comprised organizational, environmental & individual elements.  C: control group, usual work practice. | Absenteeism  Work performance (self-reported measurements for both work-related outcomes) | Workplace sitting time (objective measurement using activPAL3 activity monitor) | No significant results regarding absenteeism & work performance.  Significant decrease in sitting time at 4 week follow-up (I: mean change = -125 min / 8 h. (95% CI -161, -89; p <0.001) was replaced by standing (I: mean change = +127 min / 8 h. (95% CI +92, +162; p <0.001)). |
|  |  | Neuhaus, et al. (61)^b^ | Australia; Administrative units of the University of Queensland | N= 44 (I1=16, I2=14, C=14); Quasi-randomized controlled study; 13 weeks (baseline, 3-month follow-up) | I1: Multi-component intervention (installation of height-adjustable workstations, management consultation, staff education, manager e-mails to staff, face-to-face coaching, telephone support)  I2: height-adjustable workstations only, verbal (10-minute) and written instructions on correct usage  C: control group, advice on continuing with daily routines | Absenteeism  Work performance  (self-reported measurements) | Sitting time (objective measurements using activPAL3) | No significant changes were observed in work-related outcomes.  Significant reduction of sitting time at 3 months follow-up (I1 = 89 minutes (95% CI = -130, -47; p <0.001) compared to C and I1 = 56 minutes (95% CI = -107, -4; p = 0.033) compared to I2).  Significant increase in standing at 3 months follow-up (I1 = 93 minutes (95% CI = 45, 141; p <0.001) compared to C and I1 = 59 minutes (95% CI = 10, 107; p = 0.014) compared to I2). |
|  |  | Pedersen, et al. (41) | Denmark; 12 offices of a public administration authority | N= 549 (I1=180, I2=187, C=182); RCT; 12 months (baseline, 6-month follow-up, 12-month follow-up) | I1:Specific resistance training (SRT)  I2: All-round physical exercise (APE)  C: control group, no physical activity | Productivity (self-reported measurements using Health and Work Performance Questionnaire (HPQ)) | Physical activity, musculoskeletal pain, general health | No significant differences between the groups regarding productivity in follow-up.  No significant differences between the groups regarding physical activity in follow-up. |
|  |  | Von Thiele Schwarz and Lindfors (67) | Sweden; women working in older people’s care | N= 25 (I=13, C=12); pretest/ posttest controlled study; 12 months (baseline, 6-month follow-up, 12-month follow-up) | I: work-based PE intervention (one-hour PE sessions twice a week)  C: control group, no intervention | Productivity*  Workability*  Absenteeism*  (self-reported measurements using questionnaires) | Physical activity and physical exercise (measurements using fitness tests) | No significant differences between the groups regarding productivity, workability and absenteeism in follow-up.  No significant differences between the groups regarding fitness outcomes. |
| a: Effective intervention study on primary outcome(s)  b: Effective intervention study on secondary outcome(s)  c: Effective intervention study on primary and secondary outcomes  *: The work-related outcome(s) is/ are the primary outcome(s) of the included study | | | | | | | | |
