# Supplementary material for: The effectiveness of workplace nutrition and physical activity interventions in improving productivity, work performance and workability: a systematic review
Source: BMC Public Health. 2019 Dec 12;19:1676. doi: 10.1186/s12889-019-8033-1 (PMC6909496; doi:10.1186/s12889-019-8033-1)
Supplement: Supplementary file 3 — Additional file 3. Risk of bias in included studies. [file 12889_2019_8033_MOESM3_ESM.docx]

| Risk of Bias in included studies: low risk (+); unclear risk (?); high risk (-) | | | | | | | | | | |
| --- | --- | --- | --- | --- | --- | --- | --- | --- | --- | --- |
|  | | | Random sequence generation | Allocation concealment | Blinding of participants and personnel | Blinding of outcome assessment | Incomplete outcome data | Selective reporting | Other bias | Summary assessments |
| Environmental | Physical Activity | Ben-Ner, et al. (30)^c^ | (+) | (?) | (+) | (+) | (+) | (+) | (+) | Unclear risk of bias |
|  |  | Coffeng, et al. (57)^a^ | (+) | (+) | (+) | (+) | (+) | (+) | (+) | Low risk of bias |
| Multilevel  Organizational, Individual | Physical Activity & Nutrition | Jeffery, et al. (55) | (?) | (?) | (+) | (+) | (?) | (+) | (+) | Unclear risk of bias |
|  |  | Terry, et al. (42) | (?) | (?) | (+) | (+) | (+) | (+) | (+) | Unclear risk of bias |
|  | Physical Activity | Galinsky, et al. (31) | (?) | (?) | (+) | (+) | (+) | (+) | (+) | Unclear risk of bias |
|  |  | von Thiele Schwarz, et al. (36)^b^ | (?) | (?) | (+) | (+) | (?) | (+) | (?) | Unclear risk of bias |
|  |  | von Thiele Schwarz and Hasson (66)^a^ | (?) | (?) | (+) | (+) | (?) | (+) | (-) | High risk of bias |
|  |  | von Thiele Schwarz and Hasson (51) | (?) | (?) | (+) | (+) | (?) | (-) | (-) | High risk of bias |
| Multilevel  Environmental, Individual | Physical Activity & Nutrition | Meenan, et al. (56)^b^ | (?) | (?) | (+) | (+) | (?) | (+) | (+) | Unclear risk of bias |
|  |  | Van Dongen, et al. (63) | (+) | (+) | (+) | (+) | (+) | (+) | (+) | Low risk of bias |
|  |  | van Wier, et al. (64) | (+) | (+) | (+) | (+) | (+) | (+) | (+) | Low risk of bias |
|  | Physical Activity | Carr, et al. (37)^b^ | (+) | (+) | (+) | (+) | (+) | (+) | (+) | Low risk of bias |
|  |  | Dalager, et al. (58)^b^ | (+) | (+) | (+) | (+) | (?) | (+) | (+) | Unclear risk of bias |
|  |  | Dutta, et al. (39)^b^ | (+) | (+) | (+) | (+) | (+) | (+) | (+) | Low risk of bias |
|  |  | Jakobsen, et al. (35)^a^ | (+) | (+) | (+) | (+) | (+) | (+) | (+) | Low risk of bias |
|  |  | Puig-Ribera, et al. (32)^b^ | (+) | (?) | (+) | (+) | (+) | (+) | (+) | Unclear risk of bias |
|  |  | Snetselaar, et al. (62) | (?) | (?) | (+) | (+) | (?) | (+) | (+) | Unclear risk of bias |
| Multilevel  Environmental, Organizational, Individual | Physical Activity | Neuhaus, et al. (61)^b^ | (+) | (+) | (+) | (+) | (+) | (+) | (+) | Low risk of bias |
|  |  | Pedersen, et al. (41) | (+) | (+) | (+) | (+) | (?) | (+) | (+) | Unclear risk of bias |
| a: Effective on primary outcome(s)  b: Effective on secondary outcome(s)  c: Effective on primary and secondary outcomes | | | | | | | | | | |

| Risk Of Bias In included Non-randomized Studies - of Interventions: low risk; moderate risk; serious risk; critical risk; no information (NI) | | | | | | | | | | |
| --- | --- | --- | --- | --- | --- | --- | --- | --- | --- | --- |
|  | | | Bias due to confounding | Bias in selection of participants into the study | Bias in classification of interventions | Bias due to deviations from intended interventions* | Bias due to missing data | Bias in measurement of outcomes | Bias in selection of the reported results | Summary assessments |
| Environmental | Physical Activity | Alkhajah, et al. (29)^b^ | Low | Low | Low | NI | Low | Low | Low | Low risk of bias |
|  |  | Gao, et al. (34)^c^ | Moderate | Low | Low | NI | Low | Low | Low | Moderate risk of bias |
| Multilevel  Organizational, Individual | Physical Activity & Nutrition | Aldana, et al. (43)^a^ | Moderate | Low | Low | NI | Low | Low | Low | Moderate risk of bias |
|  |  | Lahiri and Faghri (40)^c^ | Moderate | Low | Low | NI | Low | Low | Low | Moderate risk of bias |
|  |  | Loeppke, et al. (60)^c^ | Moderate | Low | Low | NI | Low | Low | Low | Moderate risk of bias |
|  |  | Schultz, et al. (50)^a^ | Moderate | Low | Low | NI | Low | Low | Low | Moderate risk of bias |
|  | Physical Activity | Dallat, et al. (53) | Low | Low | Low | NI | Low | Low | Low | Low risk of bias |
|  |  | Hunter, et al. (54) | Low | Low | Low | NI | Low | Low | Low | Low risk of bias |
|  |  | von Thiele Schwarz, et al. (62)^c^ | Moderate | Low | Low | NI | NI | Low | NI | No Information |
|  | Nutrition | Trudeau, et al. (33) | Moderate | Low | Low | NI | Low | Low | Low | Moderate risk of bias |
| Multilevel  Environmental, Individual | Physical Activity | Chau, et al. (38)^b^ | Moderate | Low | Low | NI | Low | Low | Low | Moderate risk of bias |
| Multilevel  Environmental, Organizational, Individual | Physical Activity &Nutrition | Bertera (44)^a^ | NI | Low | Low | NI | NI | Low | Low | No Information |
|  |  | Bertera (52)^c^ | Moderate | Low | Low | NI | Low | Low | Low | Moderate risk of bias |
|  |  | Braun, et al. (45)^a^ | Moderate | Low | Low | NI | Moderate | Low | Low | Moderate risk of bias |
|  |  | Conrad, et al. (46)^a^ | Moderate | Low | Low | NI | Low | Low | Low | Moderate risk of bias |
|  |  |  | Moderate | Low | Low | NI | Low | Low | Low | Moderate risk of bias |
|  |  |  | Moderate | Low | Low | NI | Low | Low | Low | Moderate risk of bias |
|  |  | Jones, et al. (47)^a^ | Moderate | Moderate | Low | NI | Low | Low | Low | Moderate risk of bias |
|  |  | Knight, et al. (48)^a^ | Moderate | Moderate | Low | NI | NI | Low | Low | No Information |
|  |  | Maes, et al. (49) | Moderate | Low | Low | NI | Low | Low | Low | Moderate risk of bias |
|  | Physical Activity | Healy, et al. (59)^b^ | Low | Low | Low | NI | Low | Low | Low | Low risk of bias |
|  |  | Von Thiele Schwarz and Lindfors (67) | Moderate | Low | Low | NI | NI | Low | NI | No Information |
| a: Effective on primary outcome(s)  b: Effective on secondary outcome(s)  c: Effective on primary and secondary outcomes  *: The domain “Bias due to deviations from intended interventions” has been omitted from the summary assessment | | | | | | | | | | |
